# Reliable Estimation of Deterioration Levels via Late Fusion Using Multi-View Distress Images for Practical Inspection

**DOI:** 10.3390/jimaging7120273

**Published:** 2021-12-09

**Authors:** Keisuke Maeda, Naoki Ogawa, Takahiro Ogawa, Miki Haseyama

**Affiliations:** 1 Office of Institutional Research, Hokkaido University, N-8, W-5, Kita-ku, Sapporo 060-0808, Japan; 2 Graduate School of Information Science and Technology, Hokkaido University, N-14, W-9, Kita-ku, Sapporo 060-0814, Japan; naoki@lmd.ist.hokudai.ac.jp; 3 Faculty of Information Science and Technology, Hokkaido University, N-14, W-9, Kita-ku, Sapporo 060-0814, Japan; ogawa@lmd.ist.hokudai.ac.jp (T.O.); miki@ist.hokudai.ac.jp (M.H.)

**Keywords:** multi-view images, deterioration level, late fusion, attention mechanism, maintenance inspection

## Abstract

This paper presents reliable estimation of deterioration levels via late fusion using multi-view distress images for practical inspection. The proposed method simultaneously solves the following two problems that are necessary to support the practical inspection. Since maintenance of infrastructures requires a high level of safety and reliability, this paper proposes a neural network that can generate an attention map from distress images and text data acquired during the inspection. Thus, deterioration level estimation with high interpretability can be realized. In addition, since multi-view distress images are taken for single distress during the actual inspection, it is necessary to estimate the final result from these images. Therefore, the proposed method integrates estimation results obtained from the multi-view images via the late fusion and can derive an appropriate result considering all the images. To the best of our knowledge, no method has been proposed to solve these problems simultaneously, and this achievement is the biggest contribution of this paper. In this paper, we confirm the effectiveness of the proposed method by conducting experiments using data acquired during the actual inspection.

## 1. Introduction

There are many infrastructures being built around the world, and the deterioration of these structures is accelerating. The number of deteriorated infrastructures that need to be repaired is increasing. For maintaining them, engineers have been performing the inspection based on their knowledge and experience [1]. Various types of distresses (e.g., crack and corrosion) with several deterioration levels occur on infrastructures [2,3,4], and repairs of infrastructures are planned according to their levels [5]. Therefore, accurate diagnosis of the levels of distresses is important, and there is an urgent need to develop technologies to support this process. Many studies have been conducted on assisting engineers with the aim of constructing techniques to perform the maintenance inspection efficiently and accurately [6,7,8,9]. Especially, in recent years, computer vision approaches have been widely applied, construction of techniques for the automatic detection and classification of distresses [10,11] has been studied.

In order to achieve efficient support using machine learning in the maintenance inspection of infrastructures, the following two points, which are in line with the actual inspection process, must be considered. The first is the provision of the area that the machine learning model focuses on when estimating the deterioration levels. In general, the maintenance inspection is a task directly related to the safety and security of users, and therefore, the final decision is made by engineers. In order for engineers to make accurate and reliable judgments, it is necessary to provide not only the results estimated by the model but also the reason for the estimation. The second is to build a model that can make the comprehensive estimation based on multiple images taken from various angles and distances. In order to accurately judge the deterioration levels, the engineers are required to check the surrounding conditions of the distress regions and whether or not the distress is progressive. For this purpose, the engineers take multi-view images of a single inspection point from different angles and distances, and judge the final deterioration levels based on these multi-view images [12]. Therefore, it is necessary to construct a model that can estimate the deterioration level from multi-view images.

Thus, it is important to construct a model that takes into account the practical inspection process, and various studies have been conducted focusing on the above first point [13,14,15,16,17]. For example, in the literature [16], the global average pooling layer is inserted into the originally constructed model, and the class activation map proposed in the field of general object recognition [18,19,20,21] is used to reveal the regions that the model paid attention to during the estimation. In addition, in the literature [15], the map obtained by the attention mechanism is not only presented as the region of interest but also introduced into the model’s feature calculation, thereby improvement of both explanatory power and classification performance becomes feasible. Thus, the latest research can improve the reliability and accuracy of the support. However, these conventional methods assume that a single image of the distress is given, and to the best of our knowledge, no deterioration level estimation method that deals with the second point has been proposed.

In the field of image recognition and computer vision, various methods have been proposed to output a single result from multi-view data [22]. Among these fusion approaches, the late fusion is one of the most effective methods. The late fusion outputs the final result by integrating the results obtained from each data based on their reliability calculated by the model. By adopting this approach, it is expected that accurate deterioration level estimation for multi-view images becomes feasible. On the other hand, the major disadvantage of the late fusion is that if the reliability of the obtained result cannot be accurately estimated, the accuracy of the integrated result will also be degraded. Therefore, the second problem can be solved by introducing the late fusion into the model that can correctly output the reliability.

In this paper, we propose a deterioration level estimation method based on the late fusion using multi-view distress images for the practical inspection. Our method introduces the attention mechanism, which is calculated based on the region of interest of the model, and thus, the model can output the estimation results with high reliability. Therefore, accurate deterioration level estimation for multi-view images can be achieved. Furthermore, by outputting the attention map obtained by the attention mechanism, it is possible to ensure the explanatory power of the results. In other words, our method not only supports multi-view images but also provides the regions of interest of the model, thus, we can simultaneously solve two problems that should be considered in the practical inspection process. This is the biggest contribution of this paper. In the experiment, the effectiveness of the proposed method is verified by using the data obtained by engineers belonging to East Nippon Expressway Company Limited during the practical inspection.

## 2. Data Collected in Practical Inspection

**Figure 1:**
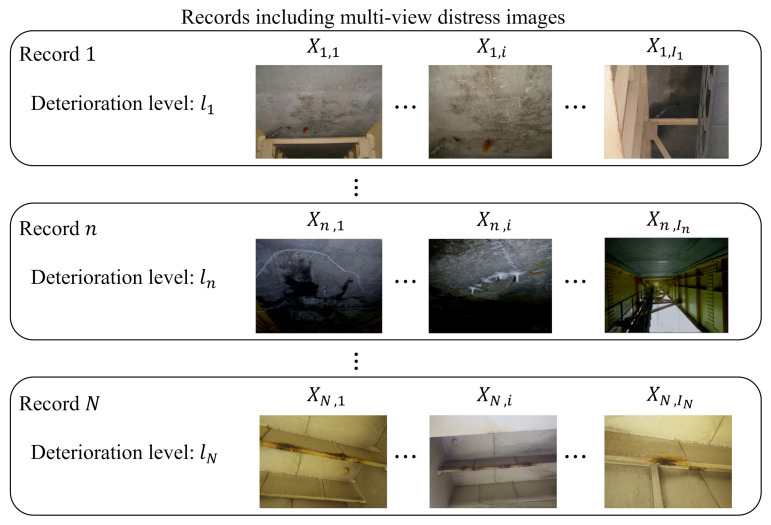
Examples of multi-view distress images taken by engineers at the practical inspection.

In this section, we explain the condition on which we focus. Engineers often take several distress images from various angles and distances, and these multi-view images are called “record”. Additionally, they determine the deterioration level for each record by referring to multi-view images. Examples of records are shown in Figure 1. As shown in this figure, we define distress images in *n*-th record as $X_{n,i_{n}}$ ($i_{n}=1,2,\ldots ,I_{n};I_{n}$ being the number of images in *n*-th record), and *N* indicates the number of records. Furthermore, the deterioration levels are denoted by $l_{n}$.

**Figure 2:**
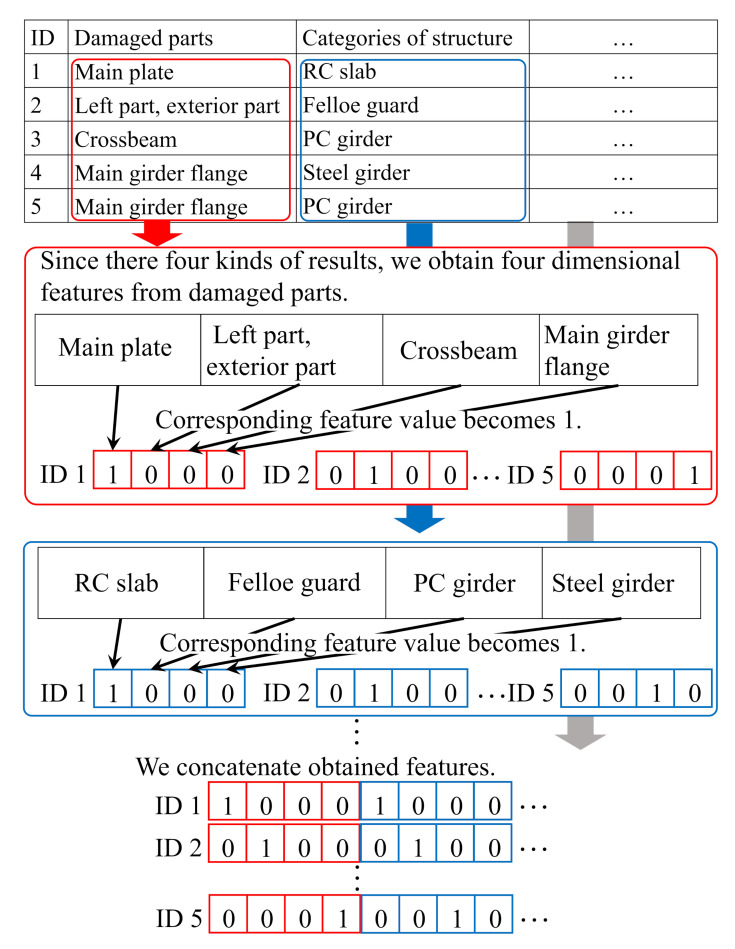
Overview of the transformation of text data into text features.

Furthermore, in the practical inspection, engineers not only take distress images but also record text data shown in Table 1. In the text data, some information related to distresses such as parts where distresses occurred, and categories of infrastructures are recorded for each distress image. Several previous studies have reported that the combination use of distress images and text data contributed to performance improvement of distress classification and deterioration level estimation [7,23,24]. Then the proposed method uses text data. In order to use text data, we have to transform these data into text features, and an overview of the procedure is shown in Figure 2. As shown in the column of “damaged parts”, since there are four kinds of results, we obtain four-dimensional features for this item. ID1 has a main plate as “damaged parts”, and the corresponding value becomes one. Otherwise, it becomes zero. We also search all items and assign the values. Finally, we obtain the text feature by concatenating the calculated features. Thus, we obtain text feature $y_{n,i_{n}}$ of the training image $X_{n,i_{n}}$ from text data.

## 3. Reliable Estimation via Late Fusion Using Multi-View Distress Images

**Figure 3:**
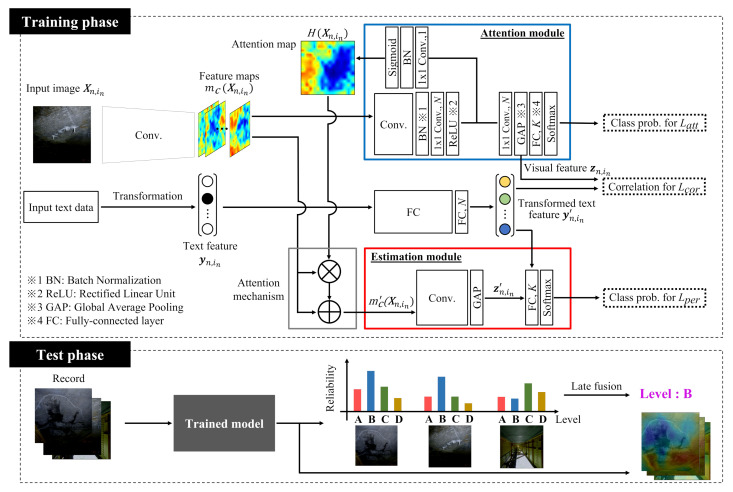
Overview of the proposed method. This model consists of the attention and estimation modules. Especially, we obtain reliable output by the estimation module when one distress image is given. Then we compare these reliabilities obtained from multi-view images, and the final estimation result with the attention map is obtained via the late fusion.

In this section, we explain the proposed method. An overview of the proposed method is shown in Figure 3. The model used in the proposed method consists of an attention module and an estimation module by referring to the previous method [15]. When one distress image and corresponding text features are input into this model, firstly the attention map can be obtained from the attention module. Furthermore, the estimation module provides reliability with the obtained attention map and feature maps in the convolutional neural network used as a backbone in the proposed method. In the test phase, when a record including multi-view images is given, we obtain reliabilities of all images. Then we average these reliabilities as the late fusion, and the final result is obtained. Note that various attention-based methods have been proposed, and especially in a recent survey paper on attention mechanisms [25], it is described that attention mechanisms can be divided into channel attention, spatial attention, and temporal attention. The contribution of this study is that the spatial attention mechanism, which can output an attention map, is applied to the field of infrastructure maintenance to improve the explanatory power of deterioration level estimation. In particular, ABN is one of reasonable and simple methods with the spatial attention mechanism since it calculates the attention map in a feed-forward operation. Therefore, we focused on ABN in this study.

### 3.1. Attention Module

The attention module generates the attention map using both features obtained from feature maps and transformed text features $y_{n,i_{n}}^{\prime }\in R^{D}$ which can be obtained through the fully connected layer. Since it has been reported in [7,24,26] that the correlation maximization is effective for deterioration level estimation, we maximize correlations between visual features from the global average pooling (GAP) layer and the transformed text features. In the conventional methods [27,28] that generate the attention map, there is the K×1×1 convolution layer before the GAP layer, and the number of dimensions of features used for calculating the correlations. Note that *K* is the number of deterioration levels. That is, the features have low expressive power and are not suitable for correlating them. Thus, by newly constructing a J×1×1 convolution layer (J≫K), this allows us to obtain visual features $z_{n,i_{n}}\in R^{J}$ with high expressive power not depending on the number of classes. Note that “K×1×1 convolution layer” represents a 1×1 kernel with *K* channels at the convolution layer. Based on the above procedures, the attention map can be calculated via feature maps transformed by passing feature maps $m_{c}\left(X_{n,i_{n}}\right)$ through the convolution layer and the rectified linear unit. Therefore, since it learns to maximize the correlation with the text data, it is expected to be able to emphasize the regions related to the distresses and lead to more reliable estimation results. This module contributes to the improvement of the interpretability.

In our attention module, the correlation between visual features and text features from the attention module is used as the loss to optimize the model. Therefore, our attention module highlights the regions where the correlation between text and image is high, i.e., the regions that are associated with distress. As a result, the attention module using multi-modal data is superior to the conventional methods such as ABN which uses only visual features.

### 3.2. Estimation Module

The generated attention map and feature maps $m_{c}\left(X_{n,i_{n}}\right)$ are input into the estimation module. Given $i_{n}$-th training image $X_{n,i_{n}}$ of *n*-th record, we calculate feature maps $m_{c}^{\prime }\left(X_{n,i_{n}}\right)$ by applying the generated attention map $H(X_{n,i_{n}})$ to the feature maps $m_{c}\left(X_{n,i_{n}}\right)$ using the attention mechanism as follows:

(1) $$m_{c}^{\prime }\left(X_{n,i_{n}}\right)=\left(1+H\left(X_{n,i_{n}}\right)\right)⊙m_{c}\left(X_{n,i_{n}}\right),$$

where ⊙ is the Hadamard product, and {c|1,2,…,C} indicates the channel. Furthermore, to achieve a performance improvement, transformed text features $y_{n,i_{n}}^{\prime }$ are also used for the final estimation. Specifically, we enable the transformed text features $y_{n,i_{n}}^{\prime }$ to be input into the fully connected layer together with $z_{n,i_{n}}^{\prime }$. As a result, $y_{n,i_{n}}^{\prime }$ and $z_{n,i_{n}}^{\prime }$ are input into a fully connected layer with a softmax function for calculating the class probabilities $p\left(X_{n,i_{n}}\right)=\left[p_{1}\left(X_{n,i_{n}}\right),\cdots ,p_{k}\left(X_{n,i_{n}}\right),\cdots ,p_{K}\left(X_{n,i_{n}}\right)\right]$. These class probabilities are regarded as the reliability used in the late fusion in the proposed method.

### 3.3. Total Loss Functions

We can train this model in an end-to-end manner based on a loss function. Specifically, the loss function in this model consists of three losses which are estimation losses from the attention and estimation modules and a correlation loss. The correlation loss can be calculated by using visual features $z_{n,i_{n}}$ and transformed text features $y_{n,i_{n}}^{\prime }$. The total loss function *L* is calculated as follows:

(2) $$L=\sum_{n}^{N}\sum_{i_{n}}^{I_{n}}\left\{L_{\mathrm{att}}\left(X_{n,i_{n}}\right)+\eta L_{\mathrm{per}}\left(X_{n,i_{n}},y_{n,i_{n}}^{\prime }\right)+\xi L_{\mathrm{cor}}\left(X_{n,i_{n}},y_{n,i_{n}}^{\prime }\right)\right\},$$

where η and ξ are the hyperparameters. $L_{\mathrm{att}}\left(X_{n,i_{n}}\right)$ and $L_{\mathrm{per}}\left(X_{n,i_{n}},y_{n,i_{n}}^{\prime }\right)$ are estimation losses from the attention and estimation modules, respectively. In the same manner as the general image classification tasks, the estimation losses are calculated by using the softmax function and cross-entropy. $L_{\mathrm{cor}}\left(X_{n,i_{n}},y_{n,i_{n}}^{\prime }\right)$, which is inspired by [29], is the loss based on the correlation between visual features and transformed text features. This is the contribution of our model. The architecture of original ABN has only $L_{att}$ and $L_{per}$, but the proposed method adds $L_{cor}$, which is different from original ABN. By introducing $L_{cor}$, the correlation between text and visual features can be maximized, and the accuracy of the attention module and estimation module can be improved. We define $z=\left[z_{1,1},\ldots ,z_{1,I_{1}},\ldots ,z_{n,I_{n}},\ldots ,z_{N,1},\ldots ,z_{N,I_{N}}\right]\in R^{J\times (I_{1}+\cdots +I_{N})}$ using visual features $z_{n,i_{n}}$ from the attention module and $Y=\left[y_{1,1}^{\prime },\ldots ,y_{1,I_{1}}^{\prime },\ldots ,y_{n,I_{n}}^{\prime },\ldots ,y_{N,1}^{\prime },\ldots ,y_{N,I_{N}}^{\prime }\right]\in R^{D\times (I_{1}+\cdots +I_{N})}$ using transformed text features $y_{n,i_{n}}^{\prime }$. In [29], the correlation objective between the two feature groups is denoted by Corr(Z,Y), and $L_{\mathrm{cor}}\left(X_{n,i_{n}},y_{n,i_{n}}^{\prime }\right)=-\mathrm{Corr}\left(Z,Y\right)$ is calculated. By training this model based on the total loss function *L*, generation of the attention map and reliable estimation of the deterioration level become feasible.

### 3.4. Final Estimation Based on Late Fusion

In the test phase, when a record including multi-view distress images $\left\{X_{1}^{\mathrm{test}},\cdots ,X_{I}^{\mathrm{test}}\right\}$ is given, we input the multi-view images into the model trained according to the above procedures explained in the previous subsections. Then we can obtain the reliabilities $p\left(X_{i}^{\mathrm{test}}\right)=\left[p_{1}\left(X_{i}^{\mathrm{test}}\right),\cdots ,p_{k}\left(X_{i}^{\mathrm{test}}\right),\cdots ,p_{K}\left(X_{i}^{\mathrm{test}}\right)\right]$ from each image. In the proposed method, by averaging the reliabilities obtained from all images, we can estimate the final class with the highest reliability as follows:

(3) $$\begin{array}{c} \arg\max_{k\in \{1,\ldots ,K\}}\left(\frac{1}{I}\sum_{i}^{I}p_{k}\left(X_{i}^{\mathrm{test}}\right)\right). \end{array}$$

Consequently, the proposed method can correctly estimate the deterioration level of the record including multi-view images. In this way, by performing the late fusion of multiple estimation results, we can deal with the record. Furthermore, as shown in the bottom right of Figure 3, we can provide the attention map obtained from the model. Therefore, our method simultaneously solves the two problems and contributes to support for the practical inspection.

## 4. Experimental Results

In this section, we show experimental results to verify the effectiveness of the proposed method. We explain experimental conditions in Section 4.1 and performance evaluation in Section 4.2.

### 4.1. Experimental Conditions

**Figure 4:**
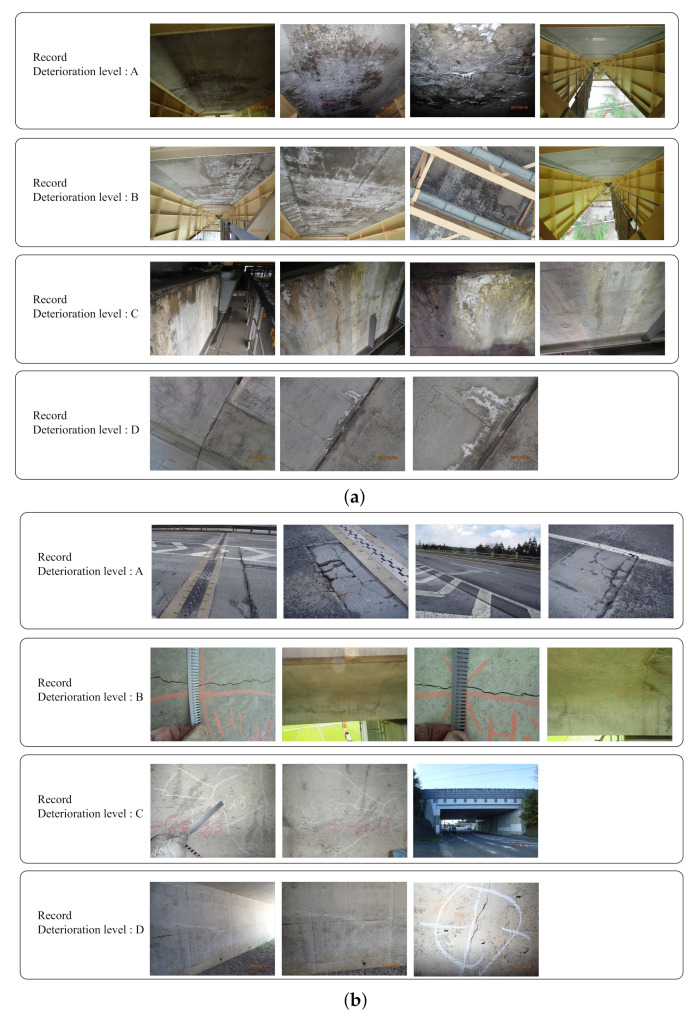
The examples of distress images in records.

The dataset used in the experiment was provided by East Nippon Expressway Company Limited. The dataset consists of records including some distress images and text data corresponding to the record. In this experiment, to verify the robustness of our method, we adopted two datasets for “efflorescence” and “crack” which commonly occur in infrastructures. The deterioration levels labeled to the records in the dataset are A, B, C, and D in descending order of the risk, i.e., K=4. The details are shown in previous study [30]. The examples of distress images are shown in Figure 4. The number of records in the dataset is shown in Table 2, and the number of distress images in the dataset is shown in Table 3. Note that robustness was confirmed by conducting the experiments with two of the frequently occurred distresses.

The dimensions of the text features $y_{n,i_{n}}$ for “efflorescence” and “crack” were 160 and 223, respectively. Additionally, the dimension of the transformed text features $y_{n,i_{n}}^{\prime }$ was eight. In the proposed method, the conv layers used in the input part, the attention and estimation modules are the same as the original ABN. On the other hand, the FC layer used to obtain the transformed text features is composed of four fully connected layers. The dimensions of each layer are 64, 32, 16, and 8, respectively. These layers have the sigmoid function as the activation function. In the inspection, various text data are recorded, such as location, date, name of the bridge, type and material. In particular, the data indicating the type and material have already been shown to be effective in classifying the distresses in previous studies. Therefore, the proposed method uses seven types of text data that represent type and material. Note that the same text data were used in both experiments for two distresses. The values of *J*, η, and ξ were experimentally set to 8, 1, and 0.2, respectively. In these experiments, the number of epochs was set to 60, and the initial learning rate was set to 0.01 and was reduced one-tenth every 20 epochs. The learning rate was set according to the literature [27], and the number of epochs was set to the value when the loss of the validation data was low. Note that, for the initial learning rate, we tried various patterns (0.1, 0.01, 0.001, and 0.0001), and the accuracy at 0.01 was the highest. The proposed method was constructed by partitioning ResNet-50 [31], which was pre-trained using ImageNet [32].

Since the novelty of this paper is a realization of deterioration level estimation for records consisting of multi-view distress images with high interpretability, we adopted some classification methods as comparative methods. Specifically, ResNet [31], which is the backbone architecture of the proposed method, was used as the baseline of classification methods. Furthermore, as mentioned in the introduction, to the best of our knowledge, there is no research that focuses on both improving interpretability and dealing with multi-view images. Therefore, we adopted the attention branch network (ABN), which is the state-of-the-art of interpretable convolutional neural networks. ABN focuses only on the improvement of interpretability via the output of the attention map. In this experiment, to evaluate the interpretability of the proposed method, we remove the late fusion function from the proposed method and evaluates the results for each image (Ours w/o LF). In addition, to confirm that the proposed method effectively deals with records containing multi-view distress images, we evaluate the results for each record. Note that, since comparative methods used in this experiment cannot deal with records, we introduce the same late fusion function as the proposed method and conduct comparison experiments.

As an evaluation index, we use F-measure of each deterioration level, and this index can be obtained as follows:

(4) $$\begin{array}{cc} F-\mathrm{measure} & =\frac{2\times \mathrm{Recall}\times \mathrm{Precision}}{\mathrm{Recall}+\mathrm{Precision}}, \end{array}$$

where

(5) $$\begin{array}{cc} \mathrm{Recall} & =\frac{\mathrm{Num}.\;\mathrm{of}\;\mathrm{correctly}\;\mathrm{estimated}\;(\mathrm{records}/\mathrm{images})}{\mathrm{Num}.\;\mathrm{of}\;\mathrm{correct}\;(\mathrm{records}/\mathrm{images})}, \end{array}$$

(6) $$\begin{array}{cc} \mathrm{Precision} & =\frac{\mathrm{Num}.\;\mathrm{of}\;\mathrm{correctly}\;\mathrm{estimated}\;(\mathrm{records}/\mathrm{images})}{\mathrm{Num}.\;\mathrm{of}\;\mathrm{all}\;(\mathrm{records}/\mathrm{images})\;\mathrm{estimated}\;\mathrm{into}\;\mathrm{each}\;\mathrm{level}}. \end{array}$$

The larger the index is, the higher the performance of the deterioration level estimation is.

### 4.2. Experimental Results

**Figure 5:**
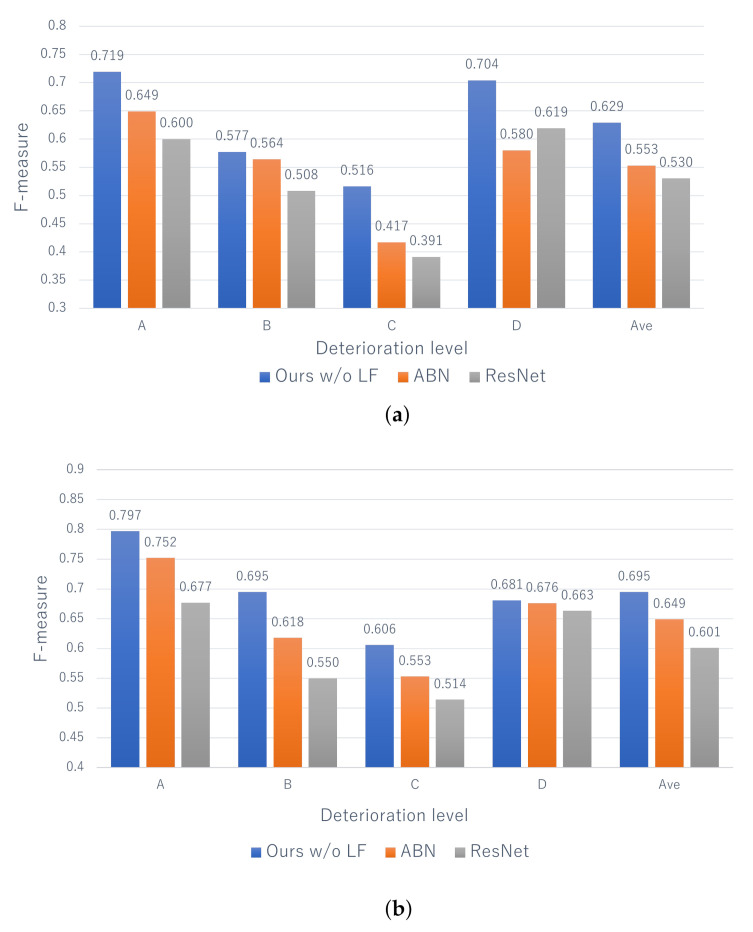
The estimation results for each image. Ours w/o LF means the proposed method without the late fusion.

Figure 5 shows the F-measure for the estimation of the deterioration levels based on the proposed method without the late fusion (Ours w/o LF) and comparative methods. It can be seen that the performance of the proposed method is better than that of comparison methods at all deterioration levels for both distresses. Specifically, the performance of the proposed method and ABN is higher than that of ResNet, and this indicates that the use of the attention map contributes to the improvement of performance. In addition, by comparing the proposed method and ABN, the effectiveness of the attention map is also clarified, which is generated by taking into account the correlation between the text data and distress images. In particular, the performance of level A, which has the highest risk, is greatly improved, and this is a significant result considering its practicality.

**Figure 6:**
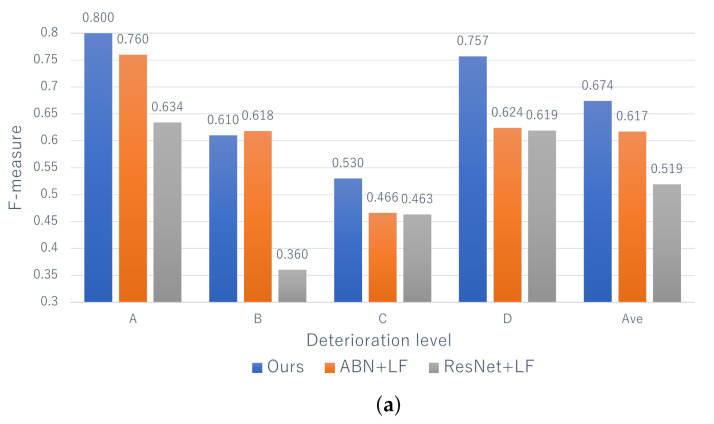

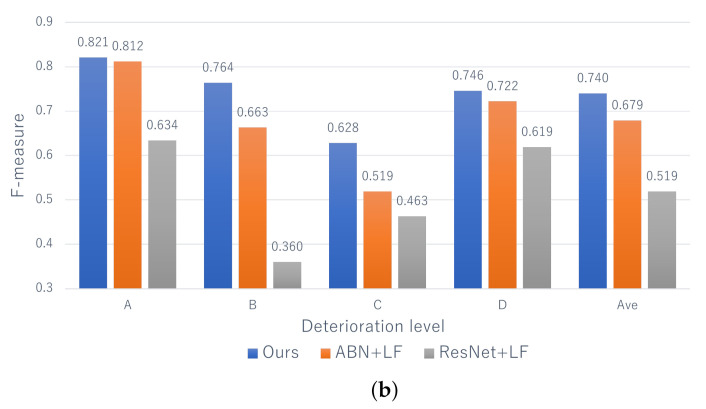
The estimation results for each record. LF indicates the late fusion.

Next, Figure 6 shows the F-measure for the estimation of the deterioration levels for each record. Since comparative methods cannot deal with the records, we applied the same late fusion approach as that of the proposed method to ABN and ResNet. Figure 6 shows that the proposed method is effective at almost all levels. Similar to the results for each image shown in Figure 5, the performance of level A, which has the highest risk, is higher than the other levels. Thus, this means that our method has a significant contribution to the practical inspection. In addition, focusing on “Ave” of the proposed method in Figure 5 and Figure 6, we can confirm the improvement of performance from 0.629 to 0.674 for efflorescence and from 0.695 to 0.740 for cracks. Therefore, it can be said that record-based estimation is more effective than image-based estimation founded on practical maintenance. In conclusion, it is clarified that the effectiveness of the proposed method for estimating the deterioration level on a record-by-record basis while maintaining high interpretability.

**Figure 7:**
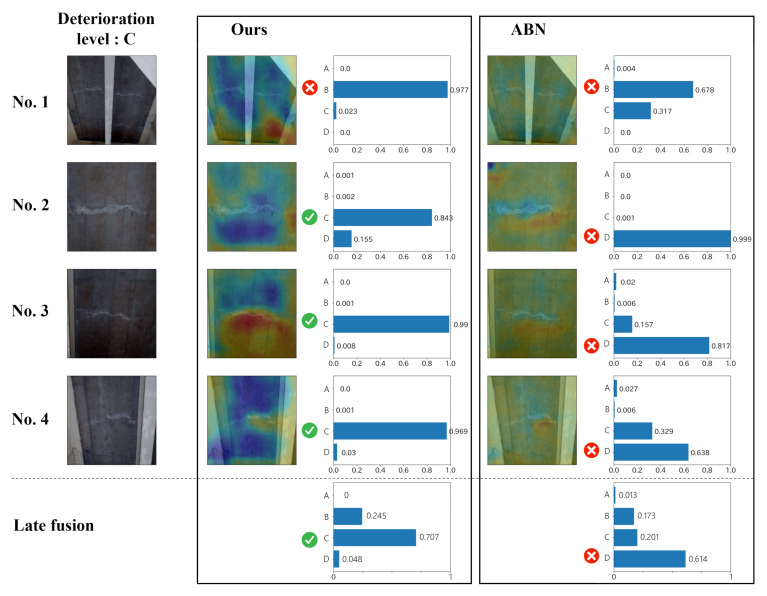
The example of estimation results of efflorescence.

**Figure 8:**
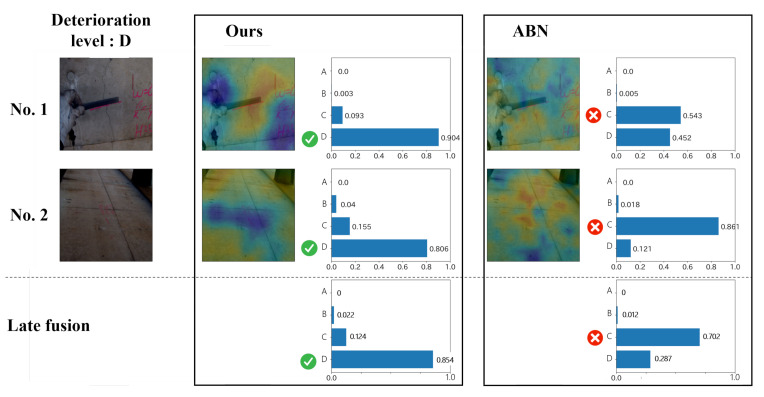
The example of estimation results of crack.

In addition, examples of the estimation results are shown in Figure 7 and Figure 8. These figures show the results of the proposed method and ABN for multi-view images contained in a single record. The images on the left side are the input for the model, and the correct deterioration level is shown above them. We show the attention map calculated by each method and the reliability for each class. From Figure 7, we can see that the attention of the ABN appears in the whole image, and this indicates that the model does not focus on the important regions. On the other hand, the proposed method, which considers the relationship between text data and images, generally focuses on the distress regions. For example, in image No. 3, we can see that the reliability is higher due to the attention to the distress regions. The bottom of the broken line in Figure 7 shows the result after the late fusion of the results for the four images above. As shown in this figure, the misestimation for image No. 1 is mitigated, and the final result becomes correct. Thus, it can be confirmed that the late fusion is effectively used for dealing with the records including multi-view images. Furthermore, Figure 8 also shows the results similar to Figure 7, but we report an interesting result for Figure 8. In a general inspection, the engineers often take a distress image of the crack while measuring the width of the crack with a ruler. As shown in image No. 1 of ABN in Figure 8, the attention appears in the area that is not related to the crack, such as a hand or a ruler. However, in the proposed method, by using text data indicating the part of distresses and materials of structures, it is possible to focus on the cracked area, as shown in the result of ours for image No. 1. In conclusion, the effectiveness of the proposed method is confirmed by its high interpretability and record-by-record estimation.

### 4.3. Discussion

In the proposed method, the average of the results obtained for multi-view images is used as the late fusion to deal with records. In approaches of the late fusion, integration based on average is one of the simplest methods, but it is necessary to consider other integration methods. Therefore, we will apply other late fusion methods as shown below to verify the performance.

- LF1 : The method adopted in the proposed method. As shown in Equation (3), we average the reliability obtained from the multi-view images and take the level with the highest value as the final estimation result.
- LF2 : In the predicted reliabilities for each deterioration level for images in the record, the deterioration level with the largest value is used as the final estimation result.
- LF3 : The deterioration level with the highest risk among the estimated levels for images in the record is used as the final estimation result.
- LF4 : The most frequently estimated level among the estimated levels for images in the record is used as the final estimation result.
- LF5 : The deterioration level estimated for a distress image randomly selected from the record is used as the final estimation result.

**Figure 9:**
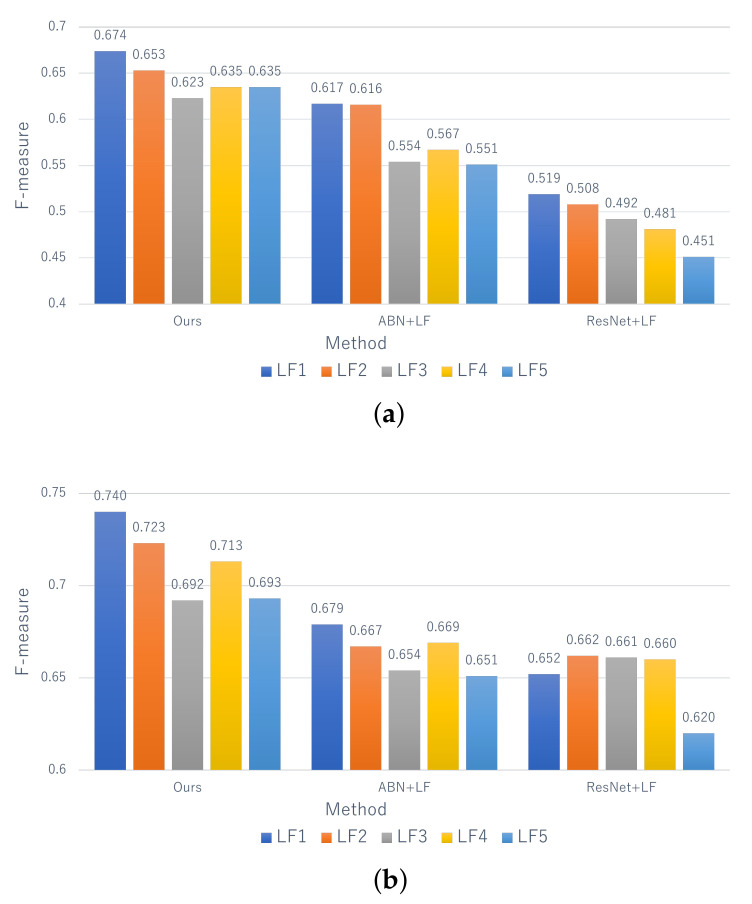
Verification of estimation performance using different late fusion.

**Figure 10:**
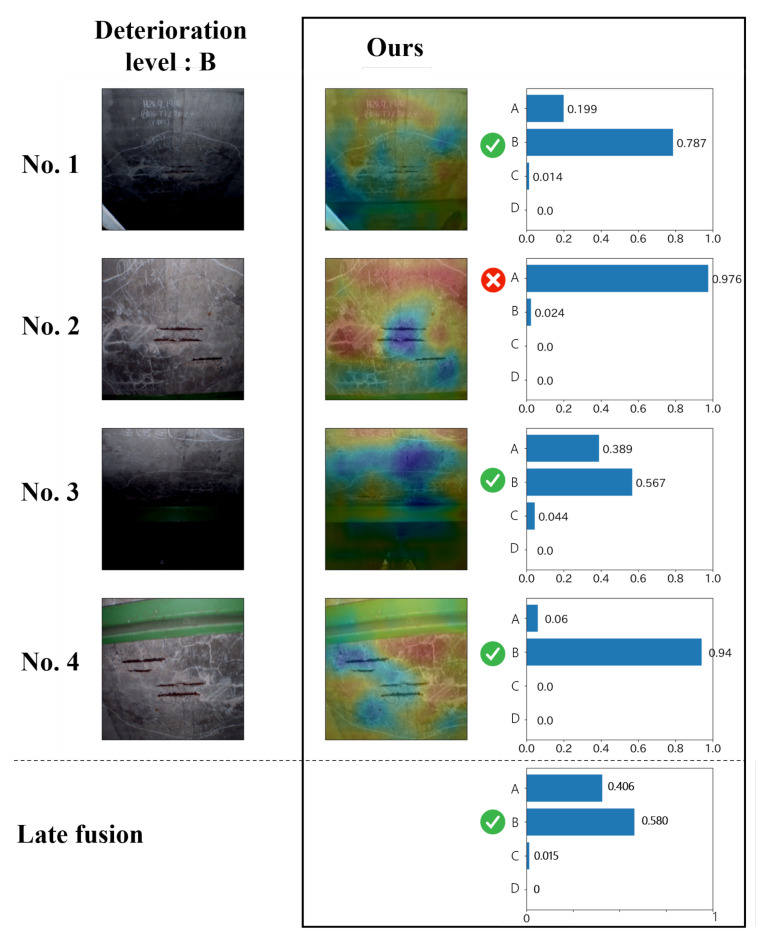
The example of the case that the most confident result is likely to be wrong.

**Figure 11:**
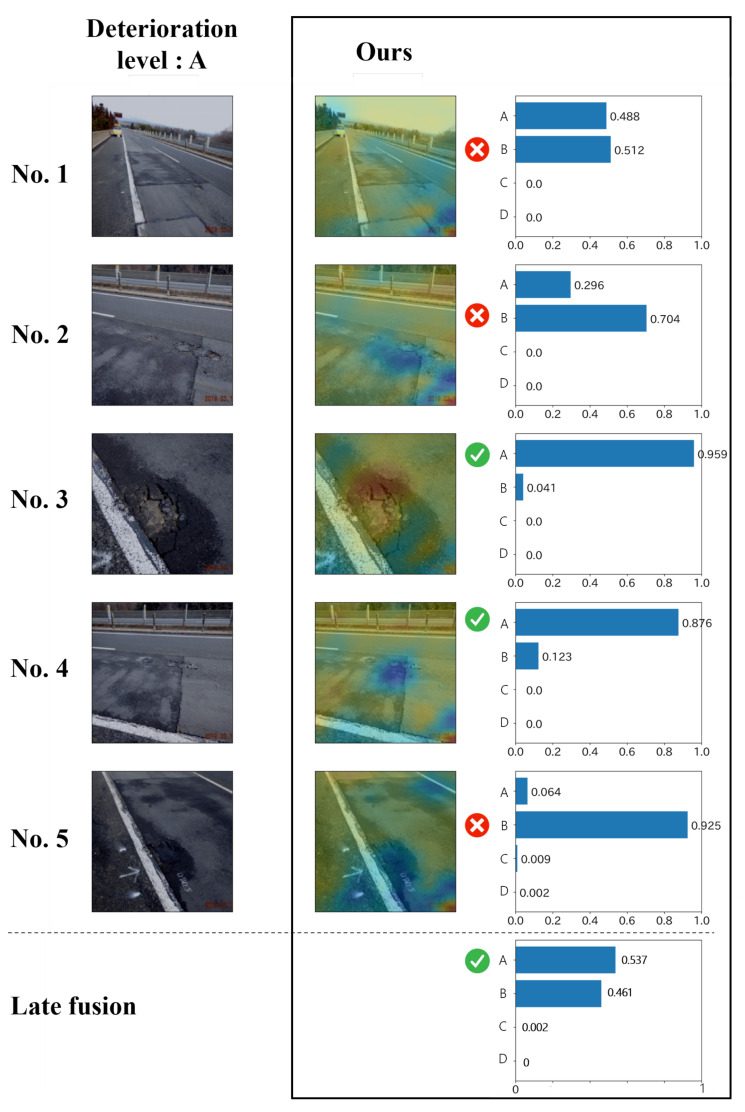
The example of the case that many images in the record are not necessarily related to the distress.

The results obtained by applying the above methods to the proposed method, ABN and ResNet are shown in Figure 9. It can be seen that the performance of LF3 and LF5 is very low for all the methods. It can be understood that not all images contribute to the estimation of deterioration levels since some images are taken during the inspection for recording the surrounding conditions of the distresses, etc. This result indicates that adopting the estimation result for a specific image is not suitable for estimating the deterioration level for a record considering the practical inspection. On the other hand, the performance of LF2 and LF4 is relatively high. However, even the most confident result is obtained such as image No. 2 in Figure 10, this result is likely to be wrong. In addition, as shown in Figure 11, LF4 is not suitable when there are many images such as images No. 1, 2, and 5 that are not directly related to the distress. From the above, it is clear that the methods LF2–5 are not suitable for actual maintenance, and LF1, which can integrate the results of all images in a record, is the most effective method.

**Figure 12:**
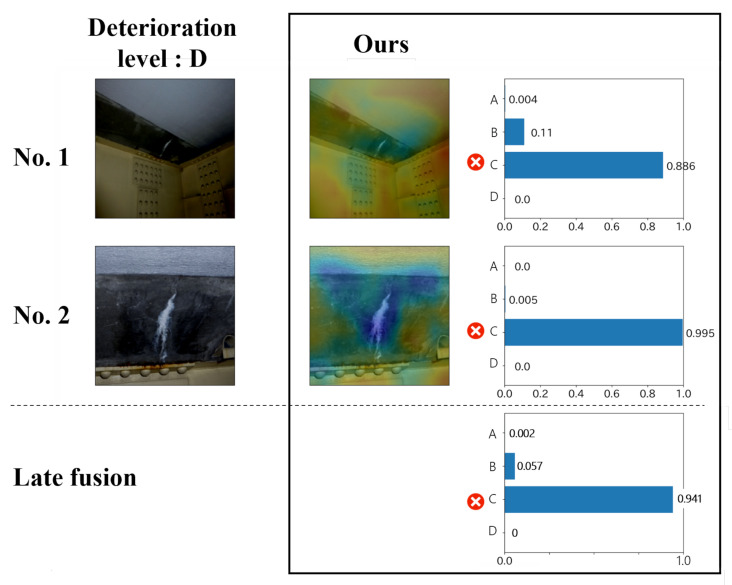
The example of incorrectly estimated results. The estimation performance for the image and record is degraded when the attention map is generated outside of the distress region.

From the above, the effectiveness of the proposed method, which has high interpretability and enables record-by-record estimation, is confirmed, but the proposed method has a performance limitation. For example, as shown in Figure 12, the estimation performance for the image and record is degraded when the attention map is generated outside of the distress region. To solve this problem, there is an urgent need to either automatically correct the attention map or establish a methodology that does not affect the performance when the attention map is not good. Since the former requires a lot of effort, the latter is more realistic, and we will discuss this approach in future work. Moreover, in this experiment, we have adopted two of the most famous distresses that occur in infrastructures. In future work, it is necessary to confirm the generalization performance of the method by using a larger number of distresses.

## 5. Conclusions

This paper has proposed a reliable estimation method of deterioration levels via the late fusion using multi-view distress images for the practical inspection. In this paper, we have simultaneously solved two problems. Specifically, we improved the interpretability of the estimation model by making it possible to generate an attention map using distress images and text data. Furthermore, based on the late fusion using the reliability obtained from the model, we have achieved highly accurate estimation for multi-view distress images. The novelty of this paper is that the above points are solved simultaneously. In the experiments, the effectiveness of the proposed method was demonstrated by comparing and verifying the results using data obtained at actual inspection sites.

As the future work, it is necessary to deal with the case where the attention map is not estimated correctly and to verify the generalization performance by using other types of distresses. Furthermore, our model can be applied to multimodal data such as images and their corresponding captions, so in future work, we will validate and extend the model using multimedia data. In addition, the effectiveness of ABN, which uses methods such as VGG and ResNext as its backbone, has been confirmed in the past literature [27]. However, since we use only ResNet as a backbone in this paper, this verification will be done in future work. Next, the late fusion approach used in the proposed method is simple. In other words, it solves the unsolved problem of practical inspection in a simple way, which is an attractive result. However, the accuracy is not sufficiently high, and some late fusion approaches have been proposed to construct multiple models and learn their reliability. Therefore, we aim to further improve the accuracy by testing various late fusion approaches in future work.

## Figures and Tables

**Table 1:** Examples of text data. Each ID corresponds to one distress image.

ID	Damaged Parts	⋯	Categories of Structure
1	Main plate	⋯	RC slab
2	Left part, exterior part	⋯	Felloe guard
3	Crossbeam	⋯	PC girder
4	Main girder flange	⋯	Steel girder
5	Main girder flange	⋯	PC girder

**Table 2:** The number of records in the dataset.

	Training				Validation				Test			
	A	B	C	D	A	B	C	D	A	B	C	D
Efflorescence	454	454	454	454	57	57	57	57	57	57	57	57
Crack	512	768	768	1024	64	96	96	128	64	96	96	128

**Table 3:** The number of distress images in the dataset.

	Training				Validation				Test			
	A	B	C	D	A	B	C	D	A	B	C	D
Efflorescence	1039	1125	1096	842	143	150	143	105	141	152	129	119
Crack	1915	2085	1897	2102	228	237	233	265	227	265	251	277

## Data Availability

Not applicable.
